# Possible regulation of genes associated with intracellular signaling cascade in rat liver regeneration

**DOI:** 10.1080/00365520802495560

**Published:** 2008-11-08

**Authors:** Cun-Shuan Xu, Heng-Yi Shao, Shuai-Shuai Liu, Bo Qin, Xiu-Feng Sun, Lin Tian

**Affiliations:** 1 College of Life Science, Henan Normal University, Xinxiang, Henan Province, P.R. China; 2 Co-Construction Key Laboratory for Cell Differentiation and Regulation, Xinxiang, Henan Province, P.R. China

**Keywords:** Genes associated with liver regeneration, intracellular signaling cascade, partial hepatectomy (PH), Rat Genome 230 2.0 array

## Abstract

**Objective:**

The importance of signal transduction in cell activities has been generally accepted. The purpose of this study was to analyze the regulatory effect of intracellular signaling cascade-associated genes on rat liver regeneration (LR) at transcriptional level.

**Material and methods:**

The associated genes were originally obtained through a search of the databases and related scientific publications; their expression profiles were then checked in rat LR using the Rat Genome 230 2.0 array. The LR-associated genes were identified by comparing the discrepancy in gene expression changes between the partial hepatectomy (PH) group and the sham operation (SO) group.

**Results:**

A total of 566 genes associated with the intracellular signaling cascade were LR related. The genes involved in nine signaling pathways including intracellular receptor-, second messenger-, nitric oxide-, hormone-, carbohydrate-mediated, protein kinase, small GTPase, ER-nuclear and target of rapamycin (TOR) signaling pathways were detected to be enriched in a cluster characterized by up-regulated expression in LR. According to their expression similarity and time relevance, they were separately classified into 5 and 5 groups.

**Conclusions:**

It is presumed that following PH, the second messenger-mediated signaling pathway inhibits the inflammatory response, while the protein kinase cascade and small GTPase-mediated signal transduction stimulate the immune response; the intracellular receptor-, second messenger-, small GTPase-mediated signal transduction and protein kinase cascade coordinately control cell replication; the intracellular receptor-, second messenger-mediated and ER-nuclear signaling pathways facilitate cell differentiation; the MAPK cascade and small GTPase-mediated signal transduction play a role in cytoskeletal reconstruction and cell migration; the second messenger-, small GTPase-mediated and IκB kinase/NFκB cascades take care of protein transport, etc., in LR.

## Introduction

Liver is unique in its ability to regenerate rapidly even in adulthood [1]. Liver regeneration (LR) is a process during which the liver recovers its mass and function after damage due to various causes such as partial hepatectomy (PH), virus infection and intoxication [1]. This regenerative process is divided into four phases including the forepart (0.5–4 h after PH), prophase (6–12 h after PH), metaphase (16–66 h after PH) and the anaphase (72–168 h after PH) according to time-course [2], and involves a series of complex physiological and biochemical activities which include cell activation, de-differentiation, proliferation and its regulation, and re-differentiation [3]. All these activities can be modulated by the actions of various signaling pathways [4,5]. These multiple signaling pathways can be roughly categorized as extracellular and intracellular signaling pathways, based on the location of signaling molecules on the cell, the latter comprising nine pathways, i.e. the intracellular receptor-, second messenger-, nitric oxide-, hormone-, carbohydrate-mediated, protein kinase, small GTPase, ER-nuclear and target of rapamycin (TOR) signaling pathways. These nine signaling pathways are not independent of each other, but are woven into a complex network by crosstalk among them, corporately governing a variety of biological processes such as cytogenesis, proliferation, differentiation, movement, apoptosis and immunity, etc [6,7]. We have previously discussed the regulatory action of cell surface receptor-mediated signal transduction pathways in rat LR [8]. To further comprehensively study the role of all the signaling pathways in LR, we investigated the expression patterns of intracellular signaling cascade-related genes in the regenerating liver following a partial (2/3) hepatectomy using the Rat Genome 230 2.0 array containing 1507 intracellular signaling cascade-related genes, confirming that 566 genes are LR associated. Based on the above data, their expression dynamics, interactions and actions during hepatic regeneration were further analyzed.

## Material and methods

### Regenerating liver preparation

The study included 276 healthy Sprague-Dawley rats (200–250 g) obtained from the Experimental Animal Center of Henan Normal University. The animals were randomly divided into 23 partial hepatectomy (PH) groups and 23 sham operation (SO) groups, with 6 rats in each group. The rats in the PH groups were subjected to an operation to remove the left lateral and median lobes of their livers, as described by Higgins & Anderson [9]. The rats were killed by cervical dislocation at 0, 0.5, 1, 2, 4, 6, 8, 12, 16, 18, 24, 30, 36, 42, 48, 54, 60, 66, 72, 96, 120, 144 and 168 h post-PHx, respectively, and their livers were instantly removed. The procured livers were immediately washed three times in phosphate buffered saline (PBS) at 4°C. For each rat, about 100–200 mg liver tissue was pooled from the middle parts of the right lobe while on ice. The liver tissues of six rats for each group (total liver mass: 0.6–1.2 g) were gathered and mixed, and then stored at −80°C until use. The SO group was subjected to the same procedure as the PH group, but without liver removal. The control for both groups was normal rat liver. In the above experiments, the animal protection laws of China were strictly enforced.

### RNA isolation and purification

Total RNA was isolated according to the Trizol reagent manual (Invitrogen Corporation, Carlsbad, Calif., USA) and then purified following the RNeasy Mini kit (Qiagen, Inc., Valencia, Calif., USA) [10,11]. RNA concentration and purity were measured using a 260/280 nm ratio [12]. The quality of total RNA samples was assessed by agarose electrophoresis (180 V, 0.5 h) with a 2:1 ratio of 28S rRNA to 18S rRNA intensities, and selected for use.

### cDNA, cRNA synthesis and purification

As a template, 5 μg total RNA was used for synthesizing the first strand of cDNA by means of SuperScript II RT (Invitrogen) and with T7-oligo dT(24) (W.M. Keck Foundation, New Haven, Conn., USA) as primer. Second-strand synthesis was carried out following the Affymetrix cDNA single-stranded cDNA synthesis kit. The resulting cDNA was purified in accordance with the cDNA purification protocol [13]; 12 μg purified cDNA subsequently served as the template for production of biotin-labeled cRNA transcript using the Gene-Chip *in vitro* transcript labeling kit (ENZO Biochemical, New York, N.Y., USA). Labeled cRNA was purified according to the cRNA purification protocol [14]. The concentration, purity and quality of cDNA and cRNA were assessed as above.

### cRNA fragmentation and microarray detection

For fragmentation, 15 μl cRNA (1 μg/μl) was incubated with 6 μl 5 × fragmentation buffer and 9 μl RNase free water for 35 min at 94°C and digested into 35–200 bp cRNA fragments. The hybridization buffer was prepared according to the Affymetrix protocol and the prehybridized Rat Genome 230 2.0 microarray was added to it. Hybridization was then carried out in a rotating chamber (60 rpm, 16 h, 45°C). After the superfluous hybridization buffer had been absorbed, the arrays were washed and stained using the GeneChip fluidics station 450 (Affymetrix Inc., Santa Clara, Calif, USA). Subsequently, they were scanned with a GeneChip Scanner 3000 (Affymetrix Inc.) and images were obtained [14].

### Microarray data analysis

The images were converted to signal value using Affymetrix GCOS 1.4 software. The probe signal values were scaled to evaluate gene expression (*p*-value < 0.05), marginal expression (0.05 < *p*-value < 0.065) and no expression (*p*-value > 0.065). Signal values of each chip were then normalized and it was evaluated whether gene expression changed according to the ratios comparing the normalized *p*-value of the PH groups with that of the control groups, e.g. ratios ≥3, up-regulated expression genes; ratios ≤0.33, down-regulated expression genes. To minimize the technical error derived from the microarray analysis, regenerating liver for each time-point was measured three times with the Rat Genome 230 2.0 microarray. Their average value was calculated for corrective value use. Finally, these values were analyzed using GeneMath, GeneSpring (Silicon Genetics, San Carlos, Calif., USA) and Microsoft Excel Software (Microsoft, Redmond, Wash., USA) [14–16].

### Identification of genes associated with liver regeneration

First, the nomenclatures of nine intracellular signaling pathways were adopted from the GENEONTOLOGY database (www.geneontology.org), and were input into the databases at NCBI (www.ncbi.nlm.nih.gov) and RGD (rgd.mcw.edu) to identify the rat, mouse and human genes associated with the intracellular signaling cascade. In addition, according to maps of biological pathways embodied by GENMAPP (www.genmapp.org), BIOCARTA (www.biocarta.com/genes/index.asp) and KEGG (www.genome.jp/kegg/pathway.html), the genes associated with the above pathways were collated and reconfirmed by a search of the literature for the pertinent articles. The genes that exhibited a greater than 3-fold change in the rat regenerating liver were referred to as meaningfully expressed genes. Besides the rat genes, the above genes that were now known only to exist in mouse and/or humans were considered as rat homologous genes. Finally, the genes that displayed the same or similar results using the three independent analyses showed meaningful expression changes in at least one time-point, and displayed a significant difference (0.01 ≤ *p* < 0.05) or an extremely significant difference (*p* ≤ 0.01) between PO and SO by F-test, were included as being associated with rat liver regeneration.

## Results

### Expression changes of intracellular signaling cascade-associated genes in rat LR

According to the information from databases such as NCBI, AMIGO, BIOCARTA, KEGG, RGD and MGI, etc., 2417 genes were involved in the intracellular signaling cascade. Among them, 110, 426, 15, 9, 2, 738, 369, 26 and 3 genes related to intracellular receptor-, second messenger-, nitric oxide-, hormone-, carbohydrate-mediated, protein kinase, small GTPase, ER-nuclear and TOR signaling pathways were found in the Rat Genome 230 2.0 array. Correspondingly, 41, 169, 6, 4, 2, 271, 138, 7 and 1 genes revealed meaningful expression changes in at least single time-points after PH, and showed significant or extremely significant differences between PH and SO, and displayed reproducible results in three independent analyses using the Rat Genome 230 2.0 array, suggesting that these genes were associated with LR. Among a total of 566 genes, 309 genes were up-expressed, 183 were down-expressed, while 74 were up-expressed at some time-points and down-expressed at others during LR (up/down-regulated for short). The range of up-regulation was 3- to 128-fold compared with the control, and that of down-regulation was 3- to 32-fold (Table I, available online at the journal website www.informa.com/gastro). Different genes varied greatly at the time-points when the expression was initiated and terminated, as well as during the persistence period of expression. In this case, the original time-point at which genes were meaningfully expressed is considered as the initially expressed time-point, thus the genes significantly altered in expression at this time-point are called initially expressed genes; we added together the numbers of genes with a 3-fold change or more at any time-point and obtained the total number of expressed genes during the whole regenerative period. The results demonstrated that initially up-regulated and down-regulated genes were 347 and 219, respectively, in LR. Specifically, the number of initially up- and down-regulated genes, orderly, involved in the above nine pathways was in the sequence 22 and 19, 97 and 71, 4 and 2, 4 and 0, 1 and 1, 173 and 98, 88 and 50, 5 and 2, 1 and 0 (Figure 1A). The total frequencies of up-regulation and down-regulation of the genes in LR were 1575 and 693, respectively, and in these nine pathways, the sequence was 92 and 63, 495 and 217, 21 and 2, 21 and 0, 2 and 1, 766 and 305, 356 and 168, 29 and 7, 2 and 0, respectively (Figure 1B).

**Figure 1:**
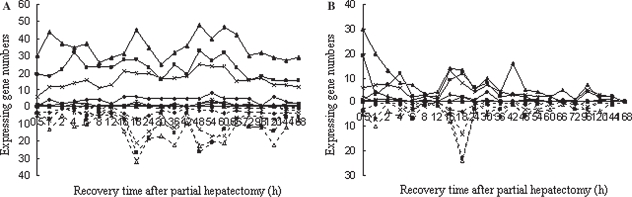
Initial and total changes in expression of 566 intracellular signaling cascade-associated genes in rat liver regeneration. The solid line denotes the up-regulated genes; the dashed line represents the down-regulated genes. A. The initial expression patterns. B. The total expression patterns. Δ = intracellular receptor-mediated signaling pathway; ▪ = second messenger-mediated signaling pathway; • = nitric oxide-mediated signaling pathway; + = hormone-mediated signaling pathway; ▪ = carbohydrate-mediated signaling pathway; ▴ = protein kinase cascade; × = small GTPase-mediated signal transduction; * = ER-nuclear signaling pathway; □ = target of rampamycin (TOR) signaling pathway.

### Expression similarity and time relevance of intracellular signaling cascade-associated genes in LR

Based on the similarity in expression, the above 556 genes were classified into the following five clusters by H-clustering analysis: only up-, predominantly up-, only down-, predominantly down-, up/down-regulation, involving 309, 19, 183, 13 and 42 genes, respectively. According to time relevance, they were categorized into 5 groups (0.5–12 h, 6 h, 16–96 h, 18–24 h and 72–144 h) and the frequencies of up-regulation and down-regulation were 407 and 89, 74 and 24, 269 and 108, 521 and 339, 304 and 133, respectively (Figure 2A). Among 58 genes up-regulated by 10-fold or more and 26 genes down-regulated by 10-fold or more, the number of up- and down-regulated genes was 3 and 2, 21 and 7, 1 and 0, 26 and 15, 12 and 5, 0 and 1, 1 and 1, in parallel, in intracellular receptor-, second messenger-, nitric oxide-mediated, protein kinase, small GTPase, ER-nuclear and general intracellular signaling pathways (Figure 2B).

**Figure 2:**
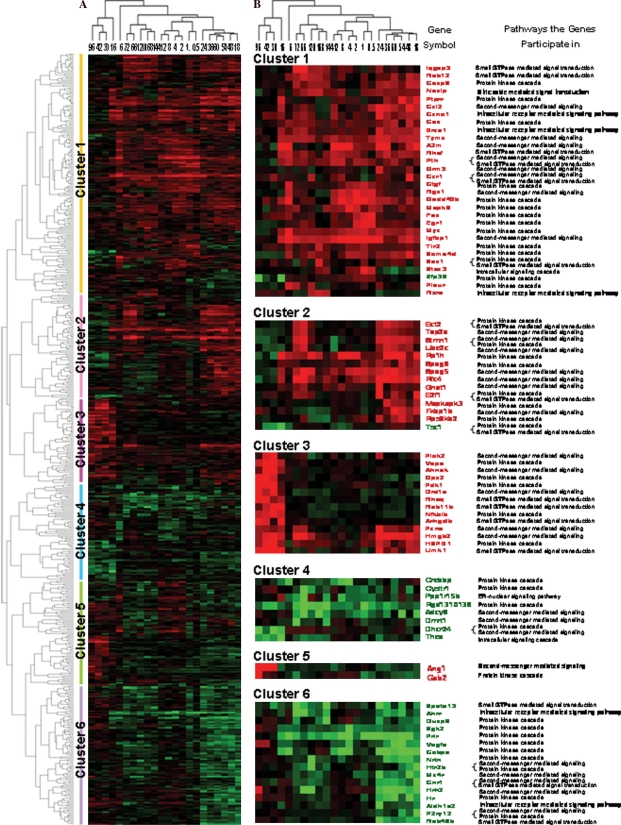
Cluster analysis of genes associated with the intracellular signaling cascade during rat liver regeneration (LR). A total of 556 genes whose intensities varied from 3-fold or over at least at one time-point in LR were subjected to H-clustering analysis. Red, black and green represent the higher, indistinctively altered and lower mRNA levels, respectively, in relation to that of control liver. The left tree and upper tree show function and time series clusters, respectively. A. Cluster assay of a total of 556 genes. B. Cluster analysis of genes with expression levels that changed 10-fold or more during LR.

### Interaction relationship among intracellular signaling cascade-associated genes in four different periods in rat LR

To answer the question of what are the interactions among the intracellular signaling cascade-related genes in the four different phases, we took advantage of the ResnetCore 1.2 software database attached in pathway studio 5.0 and constructed a network map of direct physical and transcriptional interactions between these genes. The resulting network contains 1183 genes and 3793 interactions, in which genes are depicted as greater colored spheres, and molecular relationships are represented as the physical spacing between the nodes. For convenience, here 54 representative LR-related genes were selected because of the higher level of connectivity (that is, individual genes have more than 10 interaction partners on average) and were then networked. Among the genes involved in intracellular receptor-mediated, second messenger-mediated, protein kinase cascade, small GTPase-mediated signal transduction and ER-nuclear signaling pathways, the number of up- and down-regulated genes was 3 and 3, 2 and 2, 24 and 18, 6 and 2, 1 and 1, respectively (Figure 3A). On this basis, the expression kinetics was subject to analysis. The results showed that at the forepart (0.5–4 h after PH) of LR, 14 genes were up-regulated and 8 down-regulated; at prophase (6–12 h after PH), 14 genes were up- and 3 down-regulated; at metaphase (16–66 h after PH), 25 genes were up- and 19 down-regulated; at anaphase (72–168 h after PH), 14 genes were up-regulated, 6 down-regulated and 1 up/down-regulated (Figure 3B).

**Figure 3:**
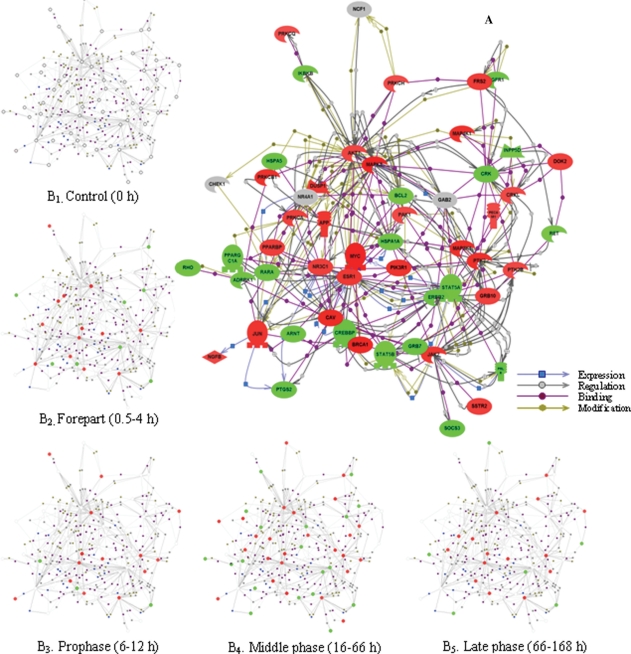
Expression dynamics and interaction of 54 intracellular signaling cascade-associated genes during rat liver regeneration (LR). The interactions of intracellular signaling cascade-associated genes were assayed by pathway studio 5.0 software. Red, green, gray and white shapes denote the up-regulated, the down-regulated, the up/down-regulated and the meaninglessly expressed genes, respectively. A. The interactions of 54 genes with closer relationships. B. Expression changes at each phase during LR.

## Discussion

The importance of signal transduction in cell activities has been generally accepted [17]. Our study demonstrated that five intracellular receptor-mediated signaling pathway-associated genes, including *ccne1* which promotes cell proliferation [18], were up-expressed during LR; while *gpr30*, which blocks cell growth and proliferation [19], was down-regulated. It was learned by a search of the peer-reviewed scientific publications that 5 genes including *sos1* involved in the small GTPase-mediated pathway, 25 genes including *igfbp1* in the second messenger-mediated pathway, 9 genes including *e2f1, hspb1* and *camkk2* in the MAPK pathway promote cell growth and division [20–22]. They were all elevated at mRNA level during LR. Notably, *sos1* was predominant at 4, 54–60 and 168 h, reaching its peak at 168 h with 15-fold of control. *igfbp1* increased in expression almost for the whole LR, and showed the expression with a marked high level of 65-fold at 1 h, basically consistent with the results reported by Crissey et al. [23]. Five genes including *rfc4* related to phosphoinositide-mediated signaling and gene *brca1* in the intracellular receptor-mediated signaling pathway are required for DNA replication and repair [24]. These genes were up-regulated mainly at metaphase; *ccnd1*, increased in expression at middle phase, has a role in cell growth and proliferation via the IκB kinase/NFκB cascade [25]. Conversely, 8 IκB kinase/NFκB cascade-related up-expressed genes including *ect2*, 2 protein kinase cascade-associated genes *gps2* and *fos* negatively control proliferation [26,27]. All 4 genes including *cnr1*, participating in the second messenger-mediated signaling pathway, and 2 MAPK cascade-involved genes (*nrtn* and *p2ry12*) stimulate neuron growth [28,29] showed significant down-regulation at the middle phase of LR. Based on the above results, it can be inferred that regenerating hepatocyte multiplication might be controlled by the above signaling pathways.

This study indicates that differentiation-promoting gene *xbp1* [30], associated with the ER-nuclear signaling pathway, was elevated in expression at 4 and 54 h post-PH, while being reduced at 144 h. In the intracellular receptor-mediated signaling pathway, 6 cell differentiation-enhancing genes containing *fhl2* and *rxra* [31] rose significantly at the middle and late stage of LR. In the second messenger-mediated signaling pathway, 6 neurogenesis and differentiation-promoting genes including *drd1a* and *ang1* were up-regulated in LR [32,33]. It is worth mentioning that *ang1* had the highest mRNA level, of 58-fold, compared with the control at 30 h. According to the above discussion, it is suggested that cell differentiation occurred mainly at the middle and late phases of LR.

Up until now, nearly all the evidence based on work with animals suggests that liver mass adjustment is precisely determined and that to some degree apoptosis may play a role during the regenerative process [2]. Research studies have shown that three MAPK cascade-associated genes play a role in induction of apoptosis [34], whereas *egr1* blocks apoptosis [35]. Among these genes, two genes, *gadd45b* and *egr1*, up-regulated mainly at middle phase, reached their highest expression abundance of 56-fold and 19-fold, respectively. In genes associated with small GTPase-mediated signal transduction, the two apoptosis-inhibiting genes *bcl6* and *ksr1* were up-expressed [36], and the apoptosis-promoting gene *dhcr24* [37] showed a trend towards decrease in expression, indicating that the above two signaling pathways have a role in the regulation of cell apoptosis.

This study revealed that 4 MAPK cascade-related genes including *stmn1* have a role in cytoskeleton organization [38]; *stmn1* was up-regulated mainly at the middle phase of LR, reaching its 16-fold peak at 66 h. Ten genes, including *iqgap3, arhgdib* and *rhof*, responsible for small GTPase Rho/Rac/Cdc42 protein signal transduction, accelerate actin cytoskeleton organization and biogenesis [39]. In all of them, mRNA levels were significantly elevated, suggesting that these two pathways positively regulate cytoskeletal rearrangement in LR. In the small GTPase-mediated signal transduction pathway, 5 up-expressed genes including *arfrp1* were essential for cell adhesion [40], probably implying that this pathway has a certain influence on adhesion of the regenerating hepatocytes and production of mechanical stress. The following cell migration-promoting genes including *crk, mapk8, ctgf* and *pak1* related to the MAPK cascade [41], *arhgdib, spata13* and *elmo1* involved in the small GTPase-mediated signal transduction pathway [42] and also *ccl2, gnat1* and *plek2* in the second messenger-mediated signaling pathway [43] were significantly enriched in the cluster characterized by gene activation post-PH, which is in accordance with the enhancement of cell migration in LR.

This work showed that in the second messenger-mediated signaling pathway, the inflammation-enhancing gene *cysltr1* [44] was down-regulated, while inflammation-attenuating *ccr1* [45] was up-regulated. Three immunoreaction-promoting genes, *sema4d, nfkbib* and *rfxank* [46–48], involved in the MAPK cascade, the NFκB cascade and small GTPase-mediated signal transduction, respectively, showed the observable rise in expression in LR. Fourteen small GTPase Rab family-associated proteins (including RAB11B, RAB12 and RAB34, etc.), which are needed for vesicular traffic and protein transport [49], were significantly up-regulated at middle and late phases of LR. Three small GTPase-mediated signal transduction-related genes including *limk1*, and IκB kinase/NFκB cascade-participating gene *vapa* were able to stimulate endocytosis [50,51] and were elevated in expression mainly at the middle phase of LR. In addition, in the retinoic acid receptor signaling pathway, the retinoic acid-catabolizing gene *cyp26b1* [52] displayed high expression abundance at 8 and 18 h. Almost in the whole LR, the down-regulated gene *prlr* inhibiting lipoprotein lipase activity [53] depending on the protein kinase cascade, fell to the lowest level of 23-fold at 0.5 h.

Taken together, the treatment of experimental material in this study is characterized by comparatively long-time and multiple time-points, and a high-throughput gene expression technique is used to investigate the expression changes and regulatory effect of genes involved in the above nine signaling pathways, post-rat PH. This facilitates investigation of the molecular mechanism of LR and gene function. After analysis, genes *igfbp1, mapk8, esr, akt* and *crk* can be preliminarily confirmed as therapeutic target candidates for liver disease. Meanwhile, this work provides a theoretical basis for studying gene therapy, selecting target genes and time-points, etc. Therefore, the above results need to be further analyzed by techniques such as protein chip, gene transfer, RNA interference, protein-interaction, and so on.
